# Cellulose Nanocomposite Hydrogels: From Formulation to Material Properties

**DOI:** 10.3389/fchem.2020.00655

**Published:** 2020-09-11

**Authors:** Svetlana Butylina, Shiyu Geng, Katri Laatikainen, Kristiina Oksman

**Affiliations:** ^1^Division of Material Science, Luleå University of Technology, Luleå, Sweden; ^2^Laboratory of Computational and Process Engineering, Lappeenranta-Lahti University of Technology, Lappeenranta, Finland; ^3^Mechanical & Industrial Engineering (MIE), University of Toronto, Toronto, ON, Canada

**Keywords:** poly(vinyl alcohol), cellulose nanocrystals, water absorption, thermograms, compression, load-unload experiments

## Abstract

Poly(vinyl alcohol) (PVA) hydrogels produced using the freeze-thaw method have attracted attention for a long time since their first preparation in 1975. Due to the importance of polymer intrinsic features and the advantages associated with them, they are very suitable for biomedical applications such as tissue engineering and drug delivery systems. On the other hand, there is an increasing interest in the use of biobased additives such as cellulose nanocrystals, CNC. This study focused on composite hydrogels which were produced by using different concentrations of PVA (5 and 10%) and CNC (1 and 10 wt.%), also, pure PVA hydrogels were used as references. The main goal was to determine the impact of both components on mechanical, thermal, and water absorption properties of composite hydrogels as well as on morphology and initial water content. It was found that PVA had a dominating effect on all hydrogels. The effect of the CNC addition was both concentration-dependent and case-dependent. As a general trend, addition of CNC decreased the water content of the prepared hydrogels, decreased the crystallinity of the PVA, and increased the hydrogels compression modulus and strength to some extent. The performance of composite hydrogels in a cyclic compression test was studied; the hydrogel with low PVA (5) and high CNC (10) content showed totally reversible behavior after 10 cycles.

## Introduction

A great number of studies were devoted to the structure and properties of PVA hydrogels. These were obtained by freeze-thawing techniques, first prepared by Peppas (1975). Despite first being prepared nearly half a century ago and commercial PVA cryogel material (Salubria™) being readily available, the study on PVA hydrogels prepared by the freeze-thawing method is still in an early stage of research. Table 1 shows the distinct research works on the PVA-based hydrogels obtained by the freeze-thaw method (or so called cryogels). The authors are aware that there are plenty of other publications on PVA-based materials produced by techniques other than freeze-thawing (e.g., injection molding, film casting, etc.) as well as methods, which include the usage of chemical cross-linking agents (for example, glutaraldehyde). However, the key interest of the authors was freeze-thaw as a method, which allows the production of three dimensional porous structures with the possibility to modify the network structure by a changing of freezing and/or thawing parameters (e.g., temperature and/or time). The formation of physical hydrogels or cross-linking through shifts in temperature is a unique feature of PVA as a synthetic polymer and it makes it similar to natural biopolymers. By means of Table 1 the authors would also like to point out that, even with the existing body of work that has already been done, there are still questions that arise when working with these materials.

**Table 1:** List of the most significant studies on pure PVA hydrogels obtained by F/T method.

**PVA**	**F/T cycles**	**Studied properties**	**References**
M_n_ = 88.8 kg/mol DP_n_ = 2,020 DH = 99.3% C = 2.5 to 15 wt.%	1 (freeze at −20°C for 45 to 120 min and thawing at 23 ± 1°C for 60 to 720 min)	Scattering light intensity of supermolecular PVA structures	Peppas, 1975
DP = 3,500 DH = 99.8% C= 5, 10 and 15 wt.%	1 to 10 (freeze at −15°C for 23 h and thaw at rt for 1 h)	X-ray diffraction, tensile properties morphology	Yokoyama et al., 1986
M_n_ = 80.0 kg/mol DH = 98.0% C = 15%	1, 2, 3, and 4 (freeze at −25°C for 16 h, and thaw at 5, 21, 35, 50°C)	Swelling, dynamic viscoelasticity	Urushizaki et al., 1990
M_n_ =35.4 kg/mol M_w_ = 79.2 kg/mol DH = 99.6% C = 10 and 15 wt.%	1 to 5 (freeze at −20°C for 1–24 h, and thaw at 23 ± 1°C for up to 24 h)	Water uptake and water diffusion in hydrogels, compressive creep experiments	Stauffer and Peppas, 1992
M_n_ = 35.74, 64.0 and 88.88 kg/mol DH = 99.0 to 99.8% C = 7, 10, 15 wt.%	3 to 7 (freeze at −20°C for 8 h and thaw at 25°C for 4 h)	Swelling and dissolution, degree of crystallinity (DSC), crystal size distribution	Hassan and Peppas, 2000; Hassan et al., 2000
Salubria™ PVA hydrogels in 0.9% saline with water content of 75 and 80%	Cyclic F/T processing	Mechanical properties, shear and compression loading	Stamen et al., 2001
M_w_ =115 kg/mol DH = 98–99% C = 11 wt.%	1 to 10 (freeze at −22°C for 20 h, and thaw at 25°C for 4 h)	Crystallinity from XRDNMR, DSC, rheological behavior	Ricciardi et al., 2003; Riccardi et al., 2004a,b
M_w_ = 95.0 kg/mol DH = 95%	15, 30 and 45 cycles (freeze at 0 °C for 8 h and thaw at 37 ± 2 °C for 16 h)	Crystallinity	Pramanick et al., 2012

As can be seen from the Table 1, it is quite difficult to compare the results of different studies because of the difference in polymer characteristics [degree of hydrolysis (DH), molecular weight (Mw), and degree of polymerization (DP)] and the difference in number and continuation of freeze and thaw (F/T) cycles. The characteristics of PVA such as degree of hydrolysis (DH), concentration (C), molecular weight (Mw and Mn), and chain tacticity all affect the gelation process (Timofejeva et al., 2017). Moreover, the importance of all these factors on properties of hydrogel were clearly demonstrated in the referred studies.

The great interest in the physically cross-linked PVA hydrogel, prepared by the freeze-thaw method, can be explained by its suitability to biomedical applications. Physically cross-linked PVA hydrogels are used in different implantable devices, for example, hydrophilic coating of catheters, tissue adhesion barriers, nerve guides, and cartilage replacements (Baker et al., 2012). Moreover, physically cross-linked PVA hydrogel has similar characteristics to soft cardiovascular tissue (Millon et al., 2006). It is also used as a drug loaded hydrogel (for example, in wound dressing) (Koehler et al., 2018).

From the other side, there is increasing interest in nanosized additives, especially in those that are bio-based, and their uses for food packaging and biomedical applications, namely drug delivery, wound dressing, and tissue engineering scaffolds (Valdés et al., 2014; Du et al., 2019; Yang et al., 2020). According to the Scopus database, the amount of publications on cellulose nanocomposite hydrogels has increased from one in 2010 to 50 in 2019, among which very few papers were devoted to physically cross-linked nanocomposite hydrogels. Nanosized cellulose is of particular interest because it enables the production of hydrogels with enhanced mechanical performance owing to its high aspect ratio and surface area, in addition to its biocompatibility and biodegradability (Curvello et al., 2019). The presence of hydroxyl groups on the surface of nano-sized cellulose is advantageous for physical cross-linking through the formation of hydrogen bonding, which in its turn is one of the current strategies in the functionalization of nanocomposite hydrogels (Tu et al., 2019). Table 2 shows examples of PVA/cellulose nanocomposite hydrogels prepared by the freeze-thaw method via physical cross-linking.

**Table 2:** PVA/cellulose nanocomposite hydrogels prepared by the freeze-thaw method.

**PVA**	**Additive**	**F/T cycles**	**Studied properties**	**References**
M_w_ = 124–186 kg/mol C = 7.5, 10, 12.5 and 15 wt.%	Bacterial cellulose 0.15 to 0.61 wt.%	1 to 6 cycles between 20 and −20°C (0.1°C/min and 1 h holding time at 20°C)	Tensile testing, stress relaxation, testing	Millon et al., 2006
M_w_ = 146–186 kg/mol DH = 99+% C = 10 wt.%	Bacterial cellulose 0, 0.30 and 0.85 wt.%	1, 3 and 6 cycles between 20 and −20°C (0.1 °C/min and 1 h holding time at-20 °C)	Unconfined compression test, stress relaxation testing	Millon et al., 2009
M_w_ = 25.0 kg/mol DH = 98.5 mol% C = 15 g PVA/100 g	CNC 0.75, 1.5, 3.0 wt.%	5 cycles (freeze at −20°C for 18 h and thaw at RT for 4 h)	Compression testing, swelling, DSC	Abitbol et al., 2011
M_w_ = 98.0 kg/mol	TEMPO CNFs 10 wt.%	1 (freeze at −18°C overnight)	Morphology, dynamic mechanical analysis (DMA)	Mihranyan, 2013
M_w_ = 146–186 kg/mol	CNC 1 wt.%	1 (freeze at −20°C for 24 h)	Compression tests, DSC, DMA	Tummala et al., 2019

The latest trend, among PVA and nanosized additives is to use the combinations of cellulose nanocrystals and chitin nanofibers (Irvin et al., 2019) or boron nitride nanosheets (Zhang et al., 2018). It has been recognized that nanosized additives affect the properties of composite material. The selection of CNC as the additive in this work was due to their numerous advantages (Pilate et al., 2016), such as low cost, availability, renewability, and exceptional physical and chemical properties.

In this study the impact of both poly(vinyl alcohol) polymer and nanocrystalline cellulose on water absorption, and thermal and mechanical properties of nanocomposite hydrogels was investigated. Pure PVA hydrogels were prepared as references. Important characteristics such as the initial water content of material prepared and their microstructures were evaluated. In addition, load/unload tests were conducted on a group of composites containing 10 wt.% of CNC.

## Materials and Methods

### Materials

**Figure 1:**
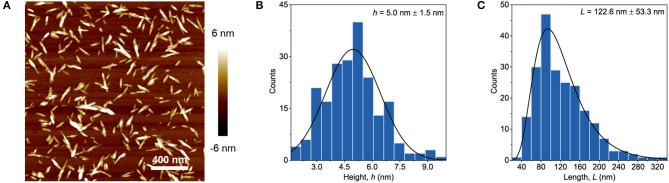
AFM image of diluted CNC sample **(A)** together with height **(B)** and length **(C)** distributions.

The commercial grade PVA which had an average molecular weight (Mw) of 89–98 kg/mol and a 99+% degree of hydrolysis was purchased from (Aldrich Chemistry). Cellulose nanocrystals (2013-FPLCNC-0499) were provided by US Forest Service, Forest Product Laboratory, Madison, USA. The product consists of 10.3 wt.% suspension of CNC in water and it also contains 1.02 wt.% sulfur determined on the base of weight of dry CNC. An AFM of highly diluted CNC dried on a mica plate was performed using a Veeco Multimode microscope with the Nanoscope V software in tapping mode. Figure 1 shows the image of the dilute suspension as well as the length and height, which were measured by using the “FibreApp” software.

### Preparation of Composite and Reference Hydrogels

The samples of the original CNC suspension were dispersed in distilled water (stirred for 24 h at 500 rpm) followed by ultrasonication by using Ultrasonic Processor UP 2005 (Hielscher Ultrasound Technology) with the 0.5 cycle and 50% amplitude for 10 min. The amount of PVA polymer listed in Table 3 was dissolved either in distilled water (for P_5_C_0_ and P_10_C_0_ reference samples) or in CNC dispersion at 90°C under intensive stirring (500 rpm) for about 3 h; the completely dissolved PVA and PVA/CNC solutions were transparent. The slightly cooled melt (~30 min kept at room temperature) was poured into a plastic mold (16 × 16 × 10 mm^3^) with 14 sections and kept there until it reached room temperature. After that, the mold was placed in the freezer at −20°C for 24 h followed by a 2 h thawing step. The formation of crosslinks between PVA chains appeared during the subsequent freeze-thawing steps. The number of freezing/thawing (F/T) cycles was selected to be three based on our previous experiments. After the last F/T cycle was over, half of each composite and each reference hydrogel samples were studied using DMA testing equipment preceded by equilibration in a distilled water bath for the period of 3 days (72 h). The other half was subjected to freeze-drying followed by different characterization procedures such as determination of water absorption, DSC, and SEM studies.

**Table 3:** Composition of investigated hydrogels.

**Sample**	**Content of PVA, w/v%**	**Content of CNC w/w (dry PVA) %**	**Number of F/T cycles**
P_5_C_0_	5	–	3
P_5_C_1_	5	1	3
P_5_C_10_	5	10	3
P_10_C_0_	10	–	3
P_10_C_1_	10	1	3
P_10_C_10_	10	10	3

### Characterization

#### Initial Water Content and Water Absorption

Initial water content is the mass of water retained in hydrogel after the manufacturing procedure has been completed and measured as the difference between the mass of hydrogel in its original state and the mass of dried hydrogel. Hydrogel samples were freeze-dried using the Alpha 2–4 LD plus a freeze dryer at a temperature of −40°C under a vacuum of 0.12 mbar for 48 h.

The freeze-dried hydrogels were then immersed in distilled water for 3 days (in the case of P_5_C_(0−10)_, hydrogels were kept up to 7 days) and their weight was measured after 1, 3, 6, 24, 48, and 72 h (a total of 168 h). Water absorption was expressed as the mass of the hydrated sample to the mass of the freeze-dried sample (g g^−1^).

#### Differential Scanning Calorimetry (DSC)

The crystalline nature of composites prepared by the freezing/thawing processes was studied using the differential scanning calorimeter, Mettler Toledo DSC821e. For the standard measurements, 5–10 mg of a freeze-dried composite sample was placed in an aluminum pan and heated at 10°C/min from 20 to 250°C.

Crystallinity was calculated according to the equation:

(1) $$\begin{array}{l} X_{c}\left(\%\right)=\frac{\Delta H_{m}-\Delta H_{cc}}{\Delta H_{m}^{\infty }}\times \frac{100}{w} \end{array}$$

where Δ*H*_m_ and Δ*H*_cc_ are the enthalpy of melting and cold crystallization of composite samples, respectively, calculated as the area of related DSC peaks, $\Delta H_{m}^{\infty }$ is the thermodynamic enthalpy of 100% crystalline PVA, which equals 138.6 J g^−1^, and w is the weight fraction of the PVA in the composite (Hassan et al., 2000; Riccardi et al., 2004a).

#### Attenuated Total Reflectance—Infrared Spectroscopy (ATR-IR)

The FTIR spectra of the freeze-dried hydrogel samples were obtained with a PerkinElmer FT-IR Spectrometer Frontier equipped with the ATR sampling accessory. Each spectrum was an average of 10 scans at resolution 4 cm^−1^ recorded in the range of 4,000 to 400 cm^−1^.

#### Compression Tests

A compression test was performed by using the submersion compression clamp on the DMA Q800 dynamic mechanical analyzer (TA Instrument). Seven square-shaped samples (16 × 16 mm^2^) with a thickness of 6 mm were tested for each type of composite. During the measurement samples were immersed in distilled water. The following testing conditions were applied in a quasi-static compression test: a compressive ramp of up to 60% strain and a strain rate of 10% per min, a preload of 0.05 N was used. In a cyclic loading/unloading test 10 cycles were continuously repeated. A relaxation time of 10 min was used in between each loading/unloading cycle.

#### Scanning Electron Microscopy (SEM)

Cross-sections were obtained by fracturing the freeze-dried samples in liquid nitrogen. The studied sample surfaces were coated prior to microscopy by using a Bal-Tec MED 020 Coating system with a tungsten target. A coating thickness of about 3–5 nm was obtained by applying a 6 × 10^−5^ mbar vacuum at 100 mA current for 20 s. A FEI Magellan 400 XHR-SEM scanning electron microscope was used.

## Results and Discussion

### Initial Water Content and Absorption of Water by Dried Hydrogels

As this research concerns hydrogels, first of all, it is important to define the content of water, because it is a main constituent of the material itself and may affect its properties (Butylina et al., 2016). As was described in the previous section, the samples of hydrogel at the end of the last (third) T/F cycle were freeze-dried. Table 4 shows the water content of the composite and reference hydrogels as well as the change in the dimensions of samples which lost their water upon drying.

**Table 4:** Water content of reference and composite hydrogels.

**Samples**	**Water content based on composition (%)**	**Water content measured (%)**	**Before and after freeze drying**
P_5_C_0_	95.00	96.37 ± 0.12	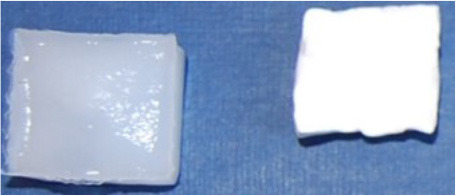
P_5_C_1_	94.95	95.56 ± 0.17	
P_5_C_10_	94.50	94.31 ± 0.27	
P_10_C_0_	90.00	91.32 ± 0.08	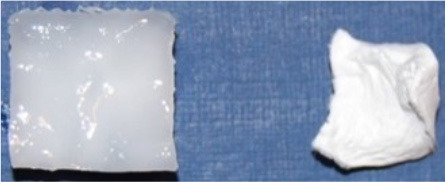
P_10_C_1_	89.90	90.30 ± 0.43	
P_10_C_10_	89.00	87.97 ± 0.23	

The water content of the P_5_C_(0−10)_ hydrogels was higher compared to the P_10_C_(0−10)_ hydrogels. The photos included in Table 4 are representative of typical photos of as-prepared and freeze-dried hydrogels belonging to the P_5_C_(0−10)_ and P_10_C_(0−10)_ groups, respectively. As it can be seen, the loss of water did not dramatically change the shape of hydrogels with formulation P_5_C_(0−10)_, it may indicate that water was uniformly distributed inside (or material was relatively homogeneous). In the case of the P_10_C_(0−10)_ hydrogels, drying led to an increase in curvature in the sample. This may indicate the heterogeneous nature or uneven distribution of water inside the P_10_C_(0−10)_ hydrogels. There are three types of water in PVA hydrogels: free water, intermediate water, and bound water (Kudo et al., 2014). The amount of free and intermediate water decreased when the cross-linking density increased which, in physically cross-linked PVA hydrogels, directly relates to the concentration of polymer (Hatakeyama et al., 2005). Moreover, according to Auriemma et al. (2006), there is a great difference in crystallization behavior in 5 and 10% PVA solutions; 5% PVA had a uniform distribution of liquid microphase.

**Figure 2:**
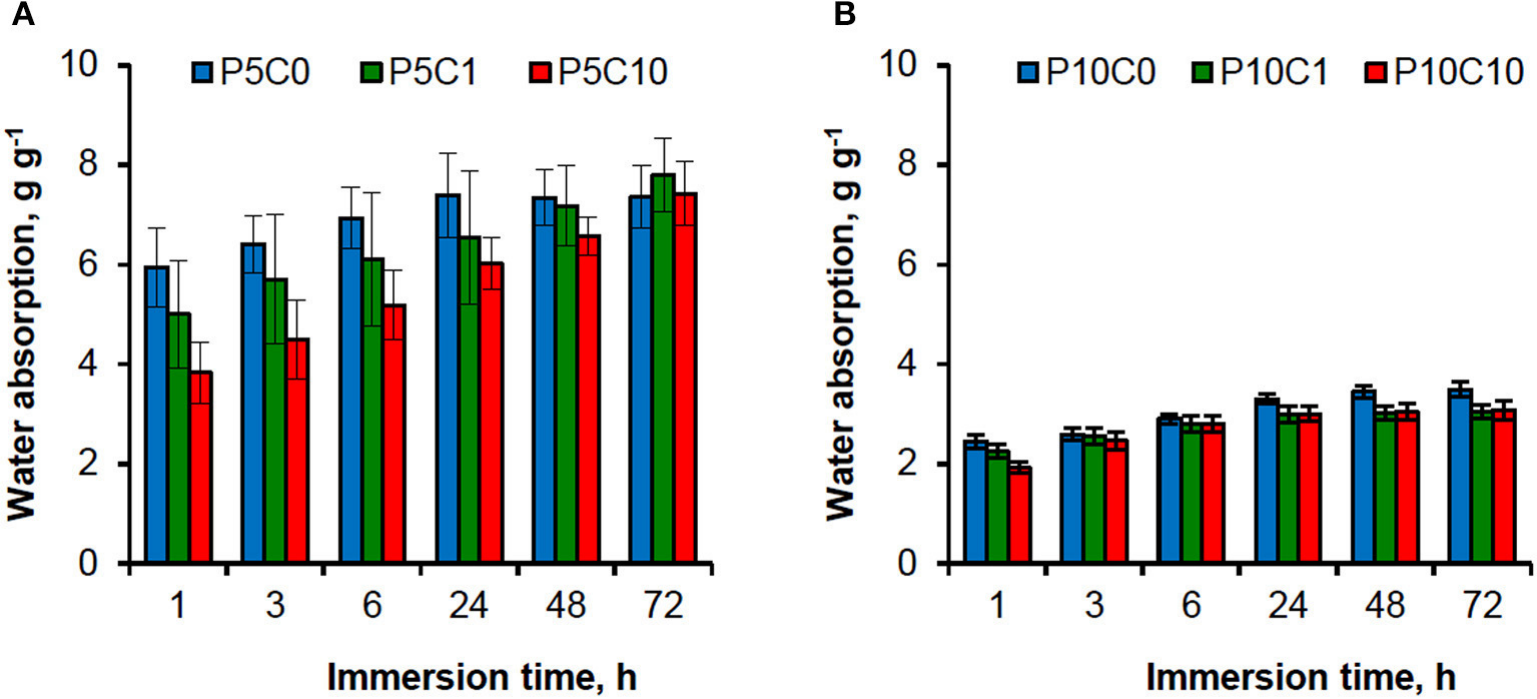
Water absorption by reference and composite hydrogels: **(A)** P_5_C_(0−10)_ and **(B)** P_10_C_(0−10)_.

The values of water absorption by composite and reference hydrogels were evaluated in a period of 72 h as presented in Figure 2. As shown, most of the water was absorbed during the first hour, after that the water absorption increased gradually. Absorption is dependent on the composition of the hydrogel, both the polymer content and CNC content affect it. The formation of denser (less porous) networks in hydrogels with higher polymer content could explain the decrease in water absorption compared to those that have lower polymer content (2.44 g g^−1^ v's 5.94 g g^−1^ after 1 h of immersion for P_10_C_0_ and P_5_C_0_, respectively). An increase in CNC concentration also led to a decrease of water absorption in composites, which was more apparent at the beginning of the immersion experiment.

The comparison of water absorption between the P_5_C_(0−10)_ and P_10_C_(0−10)_ graphs (Figure 2) revealed that the P_10_C_(0−10)_ hydrogels had a consistent level of behavior: water absorption reached its maximum level after 24 h and then was constant. In the case of the P_5_C_(0−10)_ hydrogels, similar behavior was only found for the P_5_C_0_ reference hydrogel; water absorption of the P_5_C_1_ and P_5_C_10_ composites continued to grow further and at prolonged immersion [it was monitored up to 168 h (or 7 days)] didn't reach equilibrium. Moreover, after 72 h of immersion the water absorption values of the P_5_C_1_ and P_5_C_10_ composites exceeded that of the P_5_C_0_ reference (for example, for the beforementioned samples, water absorption values measured at the end of 168 h period were 9.40 and 9.47 g g^−1^ against 8.02 g g^−1^, respectively). Earlier, Abitbol et al. (2011) showed that the minimum time for equilibrium saturation increased with an increase of CNC concentration in PVA hydrogels.

However, results obtained in this study cannot be directly compared with the study done by Abitbol et al. (2011) due to differences in the concentration and molecular weight of the PVA polymer used, as well as the dimensions of the hydrogel samples and the method of their preparation. The characteristics mentioned above may affect the porosity and crystallinity of the hydrogel, which are known to be dominating factors in swelling/absorption behavior. The crystallinity and morphology of obtained composites was studied using DSC and SEM techniques and will be discussed in the following sections.

### DSC Analysis

**Figure 3:**
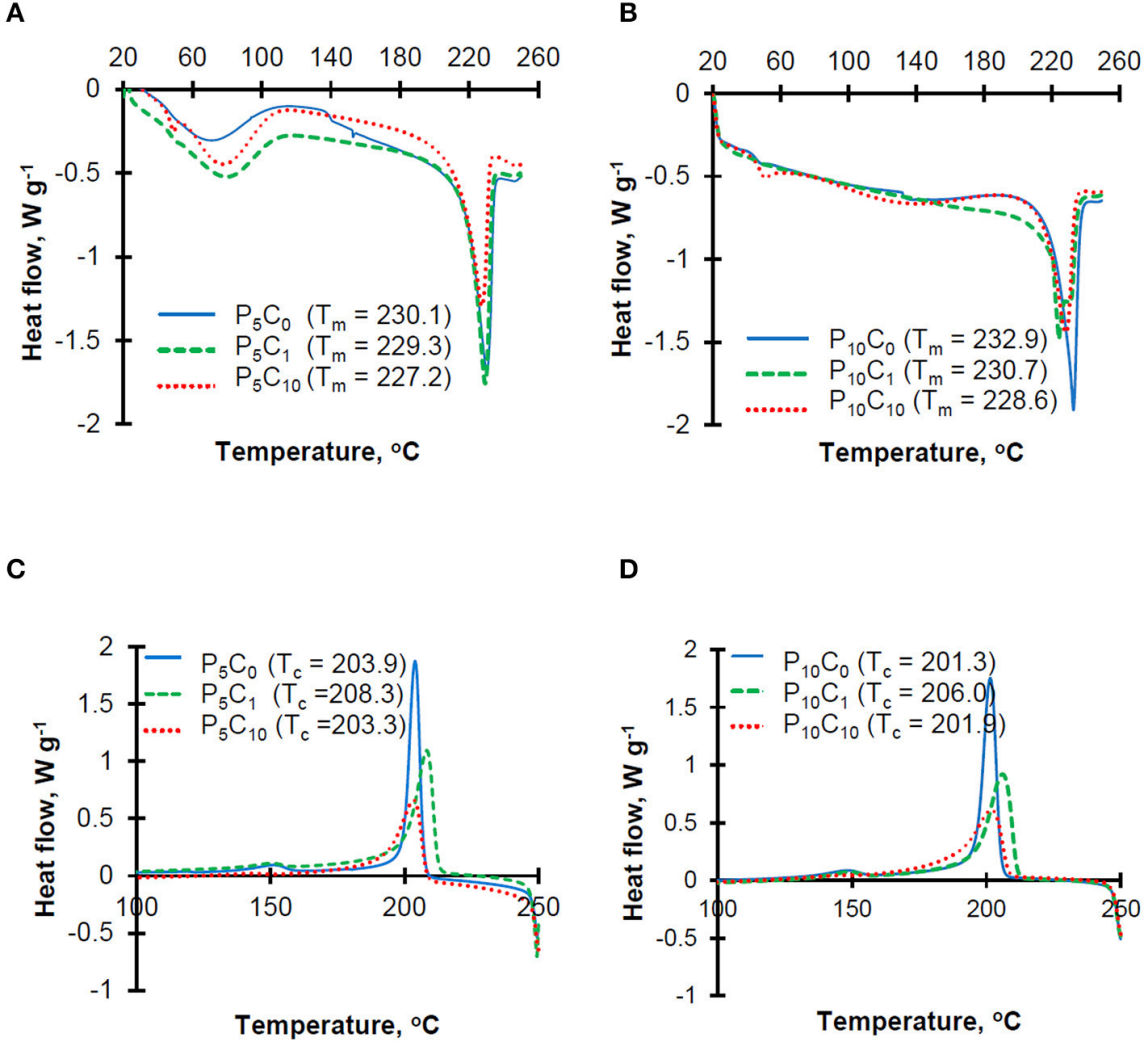
Heating **(A,B)** and cooling **(C,D)** thermograms for the reference and composite hydrogels.

Thermograms obtained at both heating and cooling stages for P_5_C_(0−10)_ and P_10_C_(0−10)_ hydrogels are shown in Figure 3.

The heating thermograms for P_5_C_(0−10)_ hydrogels (Figure 3A) were characterized by the presence of a broad peak between 60 and 100°C which represents the evaporation of residual water present in the sample. This peak was less obvious on the heating thermograms for P_10_C_(0−10)_ hydrogels (Figure 3B), which can be explained by a lower content of water in these hydrogels. The sharp peaks at ~230°C correspond to the melting point of PVA polymer. The melting temperature decreased in hydrogels with higher levels of CNC. Unusually, the heating thermogram of P_10_C_1_ composite hydrogel was characterized by the presence of a double melting peak (T_m1_ = 230.7 and T_m2_ = 224.4); this indicates the heterogeneity of crystal population. The decrease in the melting point indicates that interactions between CNC and the polymer resulted in restricted movement of the polymer chains and hindered the chain ordering that affects crystallinity. The crystallinity (*X*_”_) values obtained for hydrogels can be placed in the following order: 0.56/0.54/0.52/0.51/0.48 for the P_10_C_0_/P_5_C_0_ = P_5_C_1_/P_5_C_10_/P_10_C_1_/P_10_C_10_ samples, respectively. The reduction of crystallinity after CNC addition was more pronounced in P_10_C_(0−10)_ hydrogels. A decrease in PVA crystallinity upon the addition of different nanofillers (e.g., CNC, cellulose whiskers, and nano silica) has been discovered in earlier studies (Roohani et al., 2008; Abitbol et al., 2011; Sun et al., 2018). In theory, a reduction of crystallinity should decrease the strength of the hydrogels and increase the swelling degree (in other words water absorption) of the hydrogels. However, analysis of water absorption behavior in P_10_C_(0−10)_ hydrogels showed that despite the low crystallinity of P_10_C_1_ and P_10_C_10_ composites their water absorption values were also lower compared to the P_10_C_0_ reference, thus confirming that the relationship between the different characteristics of composites seems to be very complex. Torstensen et al. (2019) has assumed that the reduced swelling of PVA/nanocellulose composites can be linked to strong interactions between these two components as well as strong bonding between the nanocellulose itself. An effect of CNC addition on mechanical properties will be discussed in the following section.

Moreover, analysis of cooling thermograms (Figures 3C,D) revealed that the crystallization temperature of P_5_C_1_ and P_10_C_1_ composites were 4.4–4.7°C higher than the crystallization temperatures of other composite and reference samples; this means that at low concentrations CNC can act as a nucleating agent.

### ATR-IR Analysis

**Figure 4:**
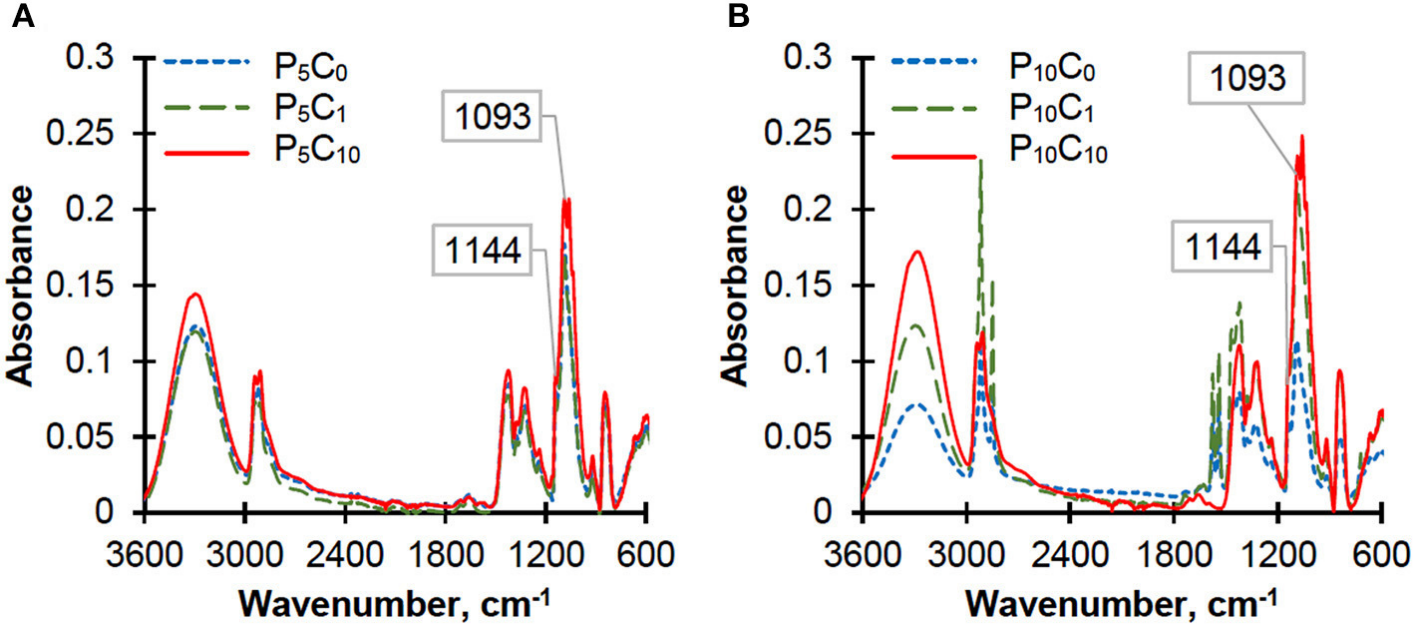
ATR-IR spectra of the reference and composite hydrogels: **(A)** P_5_C_(0−10)_ and **(B)** P_10_C_(0−10)_.

The crystallinity of PVA in reference hydrogels (P_5_C_0_ and P_10_C_0_) and in composite hydrogels was also assessed by using ATR-IR spectra (Figure 4).

According to the literature, the absorption band in the range 1,141–1,145 cm^−1^ serves as an indicator of PVA crystallinity (Mallapragada and Peppas, 1996; Tretinnikov and Zagorskaya, 2012). There are two viewpoints on the origin of this band: the first one suggests that it is associated with ν(C-O) stretching vibrations in COH groups of the crystalline phase, while the second suggests that it results from a symmetrical C-C stretching vibration within the carbon structures in the crystalline phase of PVA. The band at 1,093 cm^−1^ was used as a reference based on a proposition made by Tretinnikov and Zagorskaya (2012). They suggested that this band was most suitable as a reference due to its similarity to the 1,144 cm^−1^ band especially in cases of samples with rough surfaces, which do not allow the ideal contact with the reflecting element. The broad band at 1,093 cm^−1^ is assigned with ν(C-O) stretching vibrations. The intensity of the crystallinity band at 1,144 cm^−1^ was normalized to the intensity of the C-O stretching band at 1,093 cm^−1^ and the results can be placed in the following order: 0.539/0.466/0.460/0.440/0.439/0.388 for the P_10_C_0_/P_5_C_0_/P_5_C_1_/P_5_C_10_/P_10_C_1_/P_10_C_10_ hydrogels, respectively. As can be seen, the order based on the ATR-IR spectra is similar to the order obtained from the DSC results. However, in spite of its high sensitivity for analyzing the different structural features of polymer, this method is not suitable for direct determination of degree of crystallinity. Composite hydrogels were characterized by an increase in intensity of the following bands: the band at ~3,300 cm^−1^, which is assigned to the O-H stretching of the O-H groups both hydrogen bonded and non-hydrogen bonded, and band peak in the region of 1,093 cm^−1^ due to the contribution of C-O stretching coming from the CNC (Qua et al., 2009). The characteristic peaks at 1,055 and 1,027 cm^−1^, obtained solely in composite hydrogels, were assigned to C-O and O-C-O stretching vibrations in CNC (Abitbol et al., 2011).

### Compression Test in Quasi-Static and Cyclic Mode

In general, the stress-strain behavior of all studied hydrogels was characterized by so-called “J” shape. The initial part of the stress-strain curves corresponding to the P_5_C_(0−10)_ and P_10_C_(0−10)_ group of hydrogels showed no difference between the different samples inside the group, however, at higher strain levels, the difference between samples becomes more pronounced. Table 5 shows the data on stress and modulus of studied hydrogels obtained in quasi-stationary compression tests; the two levels of strain were chosen to evaluate the performance of hydrogels. The statistical treatment of results, which included ANOVA and Tukey's test and linear regression analysis (LRA), was carried out.

**Table 5:** Compression strength and modulus of reference and composite hydrogels evaluated at two strain values of 30 and 50 %, and a linear regression model which shows the impact of material composition on the properties.

**Sample**	**Compressive strength (kPa)**		**Compression modulus (kPa)**	
	**At 30% strain**	**At 50% strain**	**At 30% strain**	**At 50% strain**
P_5_C_0_	0.70 ± 0.12	2.14 ± 0.28	4.56 ± 0.67	10.74 ± 0.45
P_5_C_1_	0.64 ± 0.02 (NS)	2.42 ± 0.26 (NS)	4.58 ± 0.48 (NS)	15.90 ± 1.38 (S) ↑
P_5_C_10_	0.83 ± 0.17 (NS)	2.61 ± 0.37 (S) ↑	5.23 ± 0.78 (NS)	17.65 ± 3.90 (S) ↑
P_10_C_0_	5.67 ± 0.61	25.66 ± 2.81	51.74 ± 4.97	183.46 ± 14.46
P_10_C_1_	9.47 ± 0.68 (S) ↑	38.13 ± 3.16 (S) ↑	72.62 ± 5.65 (S) ↑	233.19 ± 9.88 (S) ↑
P_10_C_10_	7.34 ± 1.11 (S) ↑	29.55 ± 5.53 (NS)	56.01 ± 5.97 (NS)	150.26 ± 26.56 (S) ↓
**LRA**				
X_1_ =P,	σ_30%_ = −6.09 + 1.35 X_1_ + 0.01 X_2_		E_30%_ = −49.80 + 11.07 X_1_ – 0.20 X_2_	
X_2_ = C	σ_50%_ = −26.15 + 5.75 X_1_ – 0.05 X_2_		E_50%_ = −149.99 + 34.84 X_1_ – 2.58 X_2_	

*S corresponds to significant change (at probability level 0.05), NS corresponds to non-significant change (at probability level 0.05), and arrows show the significant trends in behavior: ↑ corresponds to improvement and ↓ corresponds to decline*.

As can be inferred from Table 5, if the directions of significant trends (marked with arrows) are scrutinized, the results are “case-sensitive.” In general, the content of the polymer predetermines the mechanical behavior of the composite, meaning that the effect of CNC was less pronounced. An increase in concentration of CNC to the level of 10% resulted in increased standard deviations (variations) and may serve as an indicator of amplified heterogeneity in gel structure. As was described in the previous section, an increase in CNC concentration from 0 to 10 wt.% resulted in a decrease in hydrogel crystallinity. As a rule, decreased crystallinity must lead to a decrease in strength, however, it was not the case here. The decrease in hydrogel strength caused by the decrease in crystallinity may have been compensated by an improved interaction and miscibility between the polymer and nanofiller (Abitbol et al., 2011).

**Figure 5:**
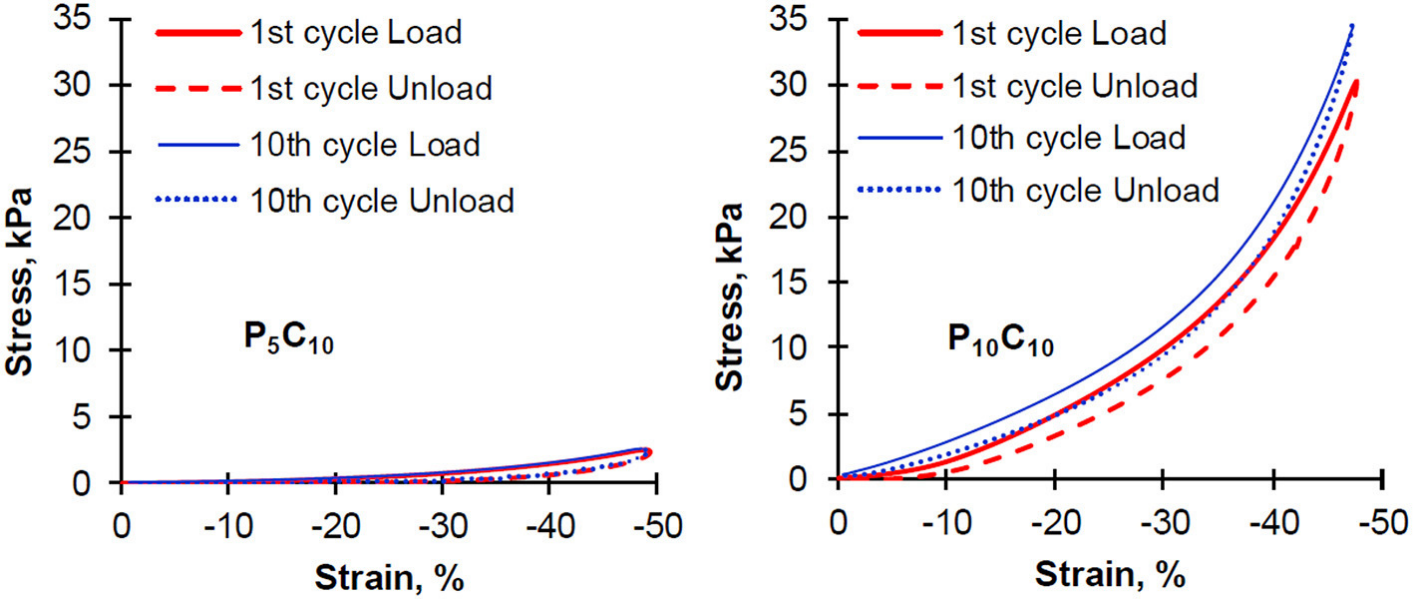
Hysteresis cycles of repeating compression experiments for P_5_C_10_ and P_10_C_10_ composite hydrogels.

To study the reversibility of composite behavior, a sequence of compression experiments consisting of 10 cycles, where load is applied and subsequently taken away, were conducted. For these experiments the P_5_C_10_ and P_10_C_10_ composites were chosen. The initial and last cycles are shown in Figure 5, the curves corresponding to other cycles were not included in the graphs to make them easily readable and all shown curves stay between the curves corresponding to the 1st and 10th cycle. Both types of composites P_5_C_10_ and P_10_C_10_ showed a hysteresis loop and the loop did not disappear even after the tenth cycle of compressive deformation. As it can be seen in Figure 5, the hysteresis loss value calculated as the inner area surrounded by the hysteresis loop were higher for P_10_C_10_ compared to P_5_C_10_ composites (84 vs. 21); the values of hysteresis loss were only slightly decreased with the increase in the number of cycles. An earlier study on the physical PVA gels showed that hysteresis was not suppressed after a repeated cyclic deformation at a reduction of 50% (Otsuka et al., 2012). Non-suppression of stress-strain hysteresis can be used as a measure of hydrogel toughness (Zhao, 2014) and there is tremendous demand for tough hydrogels in various applications (e.g., artificial loadbearing tissues, oilfield packers, hydrogel-based actuators, and soft machines).

**Figure 6:**
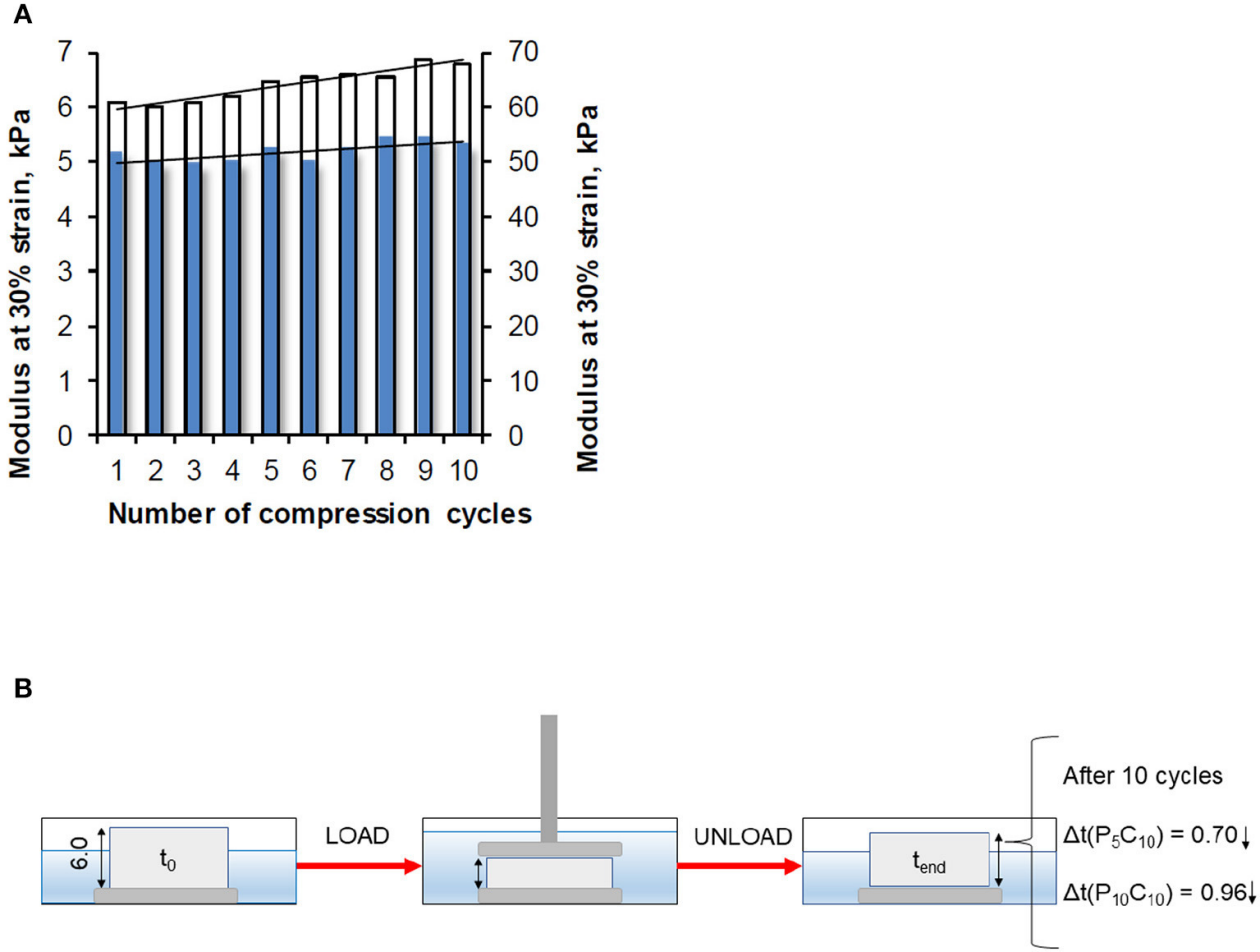
**(A)** Modulus measured at strain level 30% as a function of the number of compression cycles for the P_5_C_10_ composite (blue bars) and P_10_C_10_ composite (white bars), and **(B)** change in thickness of composites.

Modulus values of the P_5_C_10_ and P_10_C_10_ composites obtained at 30% strain as a function of the number of compression cycles are shown in Figure 6. The changes in thickness in these composites during load/unload are included. The modulus of the P_10_C_10_ composite, similar to its stress value, increased significantly with the increase in the number of applied compression cycles (from 1 to 10). This improvement is likely the result of the packaging (densification) of the structure in the P_10_C_10_ composite. As a consequence of the release of loosely bound water under compression, the decrease in the thickness of the P_10_C_10_ composite after 10 cycles was equal to 0.96 mm (14.3% of its original value). The difference in behavior of the P_5_C_10_ and P_10_C_10_ composites under cyclic compression experiments can be explained by differences in their morphology (porosity) and swelling ability. As has been demonstrated in our previous research, the stress and modulus are strongly dependent on the water content of composites.

### SEM Analysis of P_5_C_10_ and P_10_C_10_ Composites

**Figure 7:**
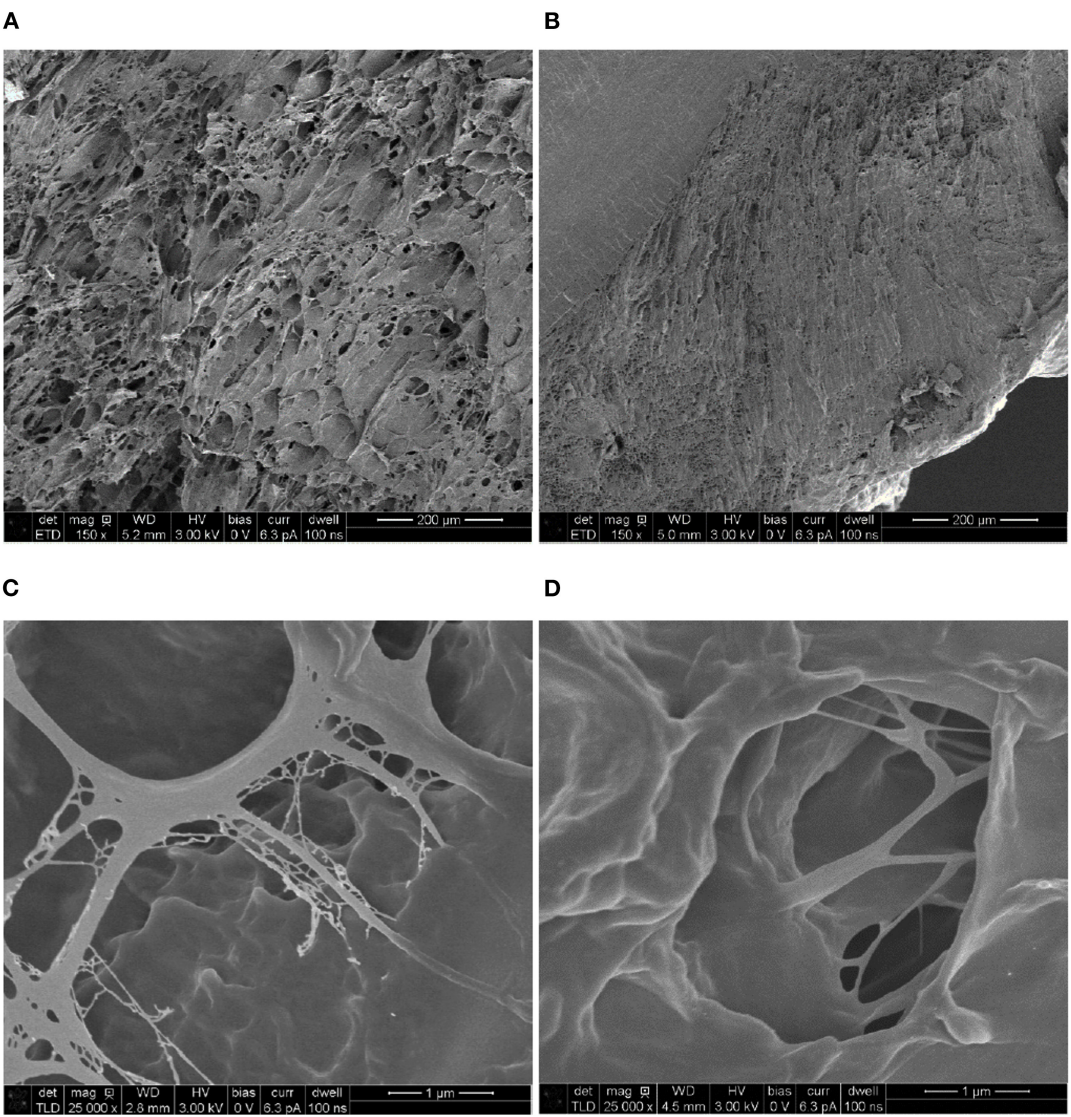
Scanning electron micrographs of composite hydrogels: **(A,C)** P_5_C_10_ and **(B,D)** P_10_C_10_.

Figure 7 shows the cross-sections of the P_5_C_10_ and P_10_C_10_ composites, which are, in general, good representatives of all hydrogels belonging to the same group: P_5_C_(0−10)_ or P_10_C_(0−10)_. As can been shown in our previous research, the content of CNC did not change the morphology of composite hydrogels, while the content of PVA polymer changed it drastically. It is clear that the size of voids (pores) in the P_10_C_10_ composite was much smaller than the size of voids in P_5_C_10_ composites, moreover all P_10_C_(0−10)_ hydrogels were characterized by the presence of dense (non-porous) areas as shown in the upper left corner of Figure 7B. Pore structure (in Figures 7C,D) was very much determined by the concentration of the polymer. The more concentrated the polymer solution was the thicker the structure of the pore's walls became. A less concentrated PVA solution caused pores to increase in size, this can be explained by the size of the water-based ice regions formed during freezing.

On the general level, the microstructure of P_5_C_(0−10)_ hydrogels was more uniform than the microstructure of P_10_C_(0−10)_ hydrogels. The non-homogeneity in hydrogel structure, as was mentioned before, can be responsible for the irregular shrinkage of P_10_C_(0−10)_ hydrogels and bend in dried samples. P_10_C_(0−10)_ hydrogels with dense structures are less prone to absorb water, on the other hand they are more rigid because water has a plasticizing effect. Moreover, the dense package of the polymer chain in the P_10_C_10_ composite makes relaxation after the withdrawal of load more difficult.

## Conclusion

In this work, the effect of both PVA polymer and cellulose nanocrystals on various properties of composite hydrogels produced by using the freeze-thaw method was investigated. We were interested to study the P_5_C_1_ and P_5_C_10_ composite hydrogels, due to the fact that most referred studies on PVA hydrogels were produced with PVA concentration of 10% or higher, and it was expected that CNC would have a reinforcing effect. As predicted, P_5_C_(0−10)_ hydrogels had a higher water content and a more porous structure than P_10_C_(0−10)_. Higher water content and a less dense polymer network seems to be the key reason for the lower compressive properties of the P_5_C_(0−10)_ hydrogels compared to P_10_C_(0−10)_ hydrogels. On the other hand, water absorption of P_5_C_(0−10)_ more than twice surpassed that of the P_10_C_(0−10)_ hydrogels. The impact of CNC was concentration-dependent as well as composite-dependent. In general, the addition of CNC caused a reduction in water content and crystallinity of the PVA. However, no increase in water absorption (swelling) or decrease in compressive properties for studied hydrogels was observed regardless of decreased crystallinity. At 1 wt.% CNC had a nucleating effect that was revealed by the increase of crystallization temperature. In the case of P_5_C_(0−10)_ hydrogels, the highest compressive modulus and strength were calculated for the P_5_C_10_ composite, but these values were 10 times lower than corresponding values for the P_10_C_10_ composite. A cyclic loading/unloading experiment showed that the P_10_C_10_ composite was less prone to reversible behavior than the P_5_C_10_ composite. This was due to the dense structure of the P_10_C_10_ composite. As reversible compressive behavior, excellent mechanical performance, and high-water content are important features in applications such as artificial tissue engineering, further research is needed to improve the performance of PVA based composites.

## Data Availability Statement

The datasets generated for this study are available on request to the corresponding author.

## Author Contributions

SB designed, performed most of experiments, and analyzed the data. SG performed the HR-SEM and AFM analyses of hydrogels. All authors contributed to writing the manuscript.

## Conflict of Interest

The authors declare that the research was conducted in the absence of any commercial or financial relationships that could be construed as a potential conflict of interest.

## References

[B1] Abitbol T. Johnstone T. Quinn T. M. Gray D. G. (2011). Reinforcement with cellulose nanocrystals of poly(vinyl alcohol) hydrogels prepared by cyclic freezing and thawing. Soft Matter 7:237379 10.1039/c0sm01172j

[B2] Auriemma F. De Rosa C. Triolo R. (2006). Slow crystallization kinetics of poly(vinyl alcohol) in confined environment during cryotropic gelation of aqueous solutions. Macromolecules 39, 9429–9434. 10.1021/ma061955q

[B3] Baker M. I. Walsh S. P. Schwartz Z. Boyan B. D. (2012). A review of polyvinyl alcohol and its uses in cartilage and orthopaedic applications. J. Biomed. Mater. Res. Part B 100B, 1451–1457. 10.1002/jbm.b.32694 22514196

[B4] Butylina S. Geng S. Oksman K. (2016). Properties of as-prepared and freeze-dried hydrogels made from poly(vinyl alcohol) and cellulose nanocrystals using freeze-thaw technique. Eur. Polym. J. 81, 386–396. 10.1016/j.eurpolymj.2016.06.028

[B5] Curvello R. Raghuwanshi V. S. Garnier G. (2019). Engineering nanocellulose hydrogels for biomedical applications. Adv. Colloid Interface Sci. 267, 47–61. 10.1016/j.cis.2019.03.002 30884359

[B6] Du H. Liu W. Zhang M. Si C. Zhang X. Li B. (2019). Cellulose nanocrystals and cellulose nanofibrils based hydrogels for biomedical applications. Carbohyd. Polym. 209, 130–144. 10.1016/j.carbpol.2019.01.020 30732792

[B7] Hassan C. M. Peppas N. A. (2000). Structure and morphology of freeze/thawed PVA hydrogels. Macromolecules 33, 2472–2479. 10.1021/ma9907587

[B8] Hassan C. M. Ward J. H. Peppas N. A. (2000). Modeling of crystal dissolution of poly(vinyl alcohol) gels produced by freezing/thawing processes. Polymer 41, 6729–6739. 10.1016/S0032-3861(00)00031-8

[B9] Hatakeyama T. Uno J. Yamada C. Kishi A. Hatakeyama H. (2005). Gel-sol transition of poly(vinyl alcohol) hydrogels formed by freezing and thawing. Thermochim. Acta 431, 144–148. 10.1016/j.tca.2005.01.062

[B10] Irvin C. W. Satam C. C. Meredith J. C. Shofner M. L. (2019). Mechanical reinforcement and thermal properties of PVA tricomponent nanocomposites with chitin nanofibers and cellulose nanocrystals. Comp. Part A 116, 147–157. 10.1016/j.compositesa.2018.10.028

[B11] Koehler J. Brandl F. P. Goepferich A. M. (2018). Hydrogel wound dressings for bioactive treatment of acute and chronic wounds. Eur. Polym. J. 100, 1–11. 10.1016/j.eurpolymj.2017.12.046

[B12] Kudo K. Ishida J. Syuu G. Sekine Y. Ikeda-Fukazawa T. (2014). Structural changes of water in poly(vinyl alcohol) hydrogel during dehydration. J. Chem. Phys. 140:044909. 10.1063/1.4862996 25669585

[B13] Mallapragada S. K. Peppas N. A. (1996). Dissolution mechanism of semicrystalline poly(vinyl alcohol) in water. J. Polym. Sci. Part B 34, 1339–46.

[B14] Mihranyan A. (2013). Viscoelsatic properties of cross-linked polyvinyl alcohol and surface-oxidized cellulose whisker hydrogels. Cellulose 20, 1369–1376. 10.1007/s10570-013-9882-x

[B15] Millon L. E. Mohammadi H. Wan W. K. (2006). Anisotropic polyvinyl alcohol hydrogel for cardiovascular applications. J. Biomed. Mater. Res. Part B Appl. Biomater. 79B, 305–311. 10.1002/jbm.b.30543 16680682

[B16] Millon L. E. Oates C. J. Wan W. (2009). Compression properties of polyvinyl alcohol-bacterial cellulose nanocomposite. J. Biomed. Mater. Res. Part B Appl. Biomater. 90B, 922–929. 10.1002/jbm.b.31364 19360889

[B17] Otsuka E. Komiya S. Sasaki S. Xing J. Bando Y. Hirashima Y. (2012). Effects of preparation temperature on swelling and mechanical properties of PVA cast gels. Soft Matter 88, 8129–8136. 10.1039/c2sm25513h

[B18] Peppas N. A. (1975). Turbidimetric studies of aqueous poly(vinyl alcohol) solutions. Makromol. Chem. 176, 3433–3440. 10.1002/macp.1975.021761125

[B19] Pilate F. Toncheva A. Dubois P. Raquez J. M. (2016). Shape-memory polymers for multiple applications in the material world. Eur. Polym. J. 80, 268–294. 10.1016/j.eurpolymj.2016.05.004

[B20] Pramanick A. K. Gupta S. Mishra T. Sinha A. (2012). Topological heterogeneity in transparent PVA hydrogels studied by AFM. Mater. Sci. Eng. C 32, 222–227. 10.1016/j.msec.2011.10.022

[B21] Qua E. H. Hornsby P. R. Sharma H. S. S. Lyons G. McCall R. D. (2009). Preparation and characterization of poly(vinyl alcohol) nanocomposites made from cellulose nanofibers. J. Appl. Polym. Sci. 113, 2238–2247. 10.1002/app.30116

[B22] Riccardi R. Auriemma F. de Rosa C. Laupretre F. (2004b). X-ray diffraction analysis of poly(vinyl alcohol) hydrogels, obtained by freezing and thawing techniques. Macromolecules 37, 1921–1927. 10.1021/ma035663q

[B23] Riccardi R. Auriemma F. Gaillet C. de Rosa C. Laupretre F. (2004a). Investigation of the crystallinity of freeze/thaw poly(vinyl alcohol) hydrogels by different techniques. Macromolecules 37, 9510–9516. 10.1021/ma048418v

[B24] Ricciardi R. Gaillet C. Ducouret G. Lafuma F. Laupretre F. (2003). Investigation of the relationships between the chain organization and rheological properties of atactic poly(vinyl alcohol) hydrogels. Polymer 44, 3375–3380. 10.1016/S0032-3861(03)00246-5

[B25] Roohani M. Habibi Y. Belgacem N. M. Ebrahim G. Karimi A. N. Dufresne A. (2008). Cellulose whiskers reinforced polyvinyl alcohol copolymers nanocomposites. Eur. Polym. J. 44, 2489–2498. 10.1016/j.eurpolymj.2008.05.024

[B26] Stamen J. A. Williams S. Ku D. N. Guldberg R. E. (2001). Mechanical properties of a novel PVA hydrogel in shear and unconfined compression. Biomaterials 22, 799–806. 10.1016/S0142-9612(00)00242-8 11246948

[B27] Stauffer S. R. Peppas N. A. (1992). Poly(vinyl alcohol) hydrogels prepared by freeze-thawing cyclic processing. Polymer 33, 3932–3936. 10.1016/0032-3861(92)90385-A

[B28] Sun J. Xu J. He Z. Ren H. Wang Y. Zhang L. (2018). Role of nano silica in supercritical CO2 foaming of thermoplastic poly(vinyl alcohol) and its effect on cell structure and mechanical properties. Eur. Polym. J. 105, 491–499. 10.1016/j.eurpolymj.2018.06.009

[B29] Timofejeva A. D'Este M. Loca D. (2017). Calcium phosphate/polyvinyl alcohol composite hydrogels: a review on the freeze-thawing synthesis approach and applications in regenerative medicine. Eur. Polym. J. 95, 547–565. 10.1016/j.eurpolymj.2017.08.048

[B30] Torstensen J. Ø. Helberg R. M. L. Deng L. Gregersen Ø. W. Syverud K. (2019). PVA/nanocellulose nanocomposite membranes for CO_2_ separation from flue gas. Int. J. Grenh. Gas Control 81:93102 10.1016/j.ijggc.2018.10.007

[B31] Tretinnikov O. N. Zagorskaya S. A. (2012). Determination of the degree of crystallinity of poly(vinyl alcohol) by FTIR spectroscopy. J. Appl. Spectrosc. 79, 521–526. 10.1007/s10812-012-9634-y

[B32] Tu Y. Chen N. Li C. Liu H. Zhu R. Chen S.. (2019). Advances in injectable self-healing biomedical hydrogels. Acta Biomater. 90, 1–20. 10.1016/j.actbio.2019.03.057 30951899

[B33] Tummala G. K. Bachi I. Mihranyan A. (2019). Role of solvent on structure, viscoelasticity, and mechanical compressibility in nanocellulose-reinforced poly(vinyl alcohol) hydrogels. J. Appl. Polym. Sci. 136:47044 10.1002/app.47044

[B34] Urushizaki F. Yamaguchi H. Nakamura K. Numajiri S. Sugibayashi K. Morimoto Y. (1990). Swelling and mechanical properties of poly(vinyl alcohol) hydrogels. Int. J. Pharm. 58, 135–142. 10.1016/0378-5173(90)90251-X

[B35] Valdés A. Mellinas A. C. Ramos M. Garrigós M. C. Jiménez A. (2014). Natural additives and agricultural wastes in biopolymer formulations for food packaging. Front. Chem. 2:6. 10.3389/fchem.2014.00006 24790975 PMC3982572

[B36] Yang X. Biswas S. K. Yano H. Abe K. (2020). Fabrication of ultrastiff and strong hydrogels by *in situ* polymerization in layered cellulose nanofibers. Cellulose 27, 693–702. 10.1007/s10570-019-02822-1

[B37] Yokoyama F. Masada I. Shimamura K. Ikawa T. Monobe K. (1986). Morphology and structure of highly elastic poly(vinyl alcohol) hydrogel prepared by repeated freezing-and-melting. Colloid Polym. Sci. 264, 595–601. 10.1007/BF01412597

[B38] Zhang J. Lei W. Chen J. Liu D. Tang B. Li J. (2018). Enhancing the thermal and mechanical properties of polyvinyl alcohol (PVA) with boron nitride and cellulose nanocrystals. Polymer 148, 101–108. 10.1016/j.polymer.2018.06.029

[B39] Zhao X. (2014). Multi-scale multi-mechanism design of tough hydrogels: building dissipation into stretchy networks. Soft Matter 10, 672–687. 10.1039/C3SM52272E 24834901 PMC4040255

