# Supplementary material for: Cellulose Nanocomposite Hydrogels: From Formulation to Material Properties
Source: Front Chem. 2020 Sep 11;8:655. doi: 10.3389/fchem.2020.00655 (PMC7517874; doi:10.3389/fchem.2020.00655)
Supplement: Supplementary file 1 [file Data_Sheet_1.pdf]

Supplementary Material

Cellulose nanocomposite hydrogels: From formulation to material properties

Svetlana Butylina[1,2], Shiyu Geng[1], Katri Laatikainen[2] and Kristiina Oksman[1,3*]

[1]Division of Material Science, Luleå University of Technology, SE-97187 Luleå, Sweden

[2]Laboratory of Computational and Process Engineering, Lappeenranta-Lahti University of Technology, FI-53851 Lappeenranta, Finland

[3]Mechanical & Industrial Engineering (MIE), University of Toronto, Toronto, ON, M5S 3G8, Canada

*** Correspondence:**

Corresponding Author (Kristiina Oksman)

Kristiina.Oksman@ltu.se

1 Formation of Hydrogel

Additional experiments were conducted to demonstrate the mechanism of hydrogel formation and give some idea about hydrogel stability. Due to the fact that formation of hydrogel (or physical crosslinking) upon freezing-melting process is unique intrinsic properties of poly(vinyl alcohol), the experiments were made with pure PVA without addition of nanocrystalline cellulose, CNC. Authors believe that CNC does not in principal change anything in basics of mechanisms, except that formation of crystallites (in joint points of closely placed polymer chains) may be affected by presence of foreigner particle (impurity) such as CNC.

As earlier as 1986, Yokoyama et al. in their study on PVA, have mentioned the unique property of this polymer to form hydrogels with rubber-like elasticity through the “repeated freezing-and-melting” method. The hydrogel formation can be seen in Supplementary Fig.1, as can be seen each new freezing time changes appearance of gel. The gel, which was not exposed to any freezing was kept for more than one week in a closed Petri dish (to prevent evaporation of water) and at the end was found still be in a clear “liquid-like” state (Supplementary Fig.2). It means no crosslinking is formed. Freeze-thawing (FT) causes the transformation of clear solution to opaque (turbid) dense gel; the density and turbidity is enhanced with every cycle applied.

Predictably, the more concentrated 10% PVA solution makes denser hydrogel compared to the 5% PVA solution. Segregation of PVA-rich and PVA-poor phases is especially noticeable after the first freezing; PVA-rich phase is opaque and PVA-poor phase is clear; distribution of phases is random. PVA chains in PVA-rich phase come closer and closer together while concentration of PVA

increasing and joints formed. According to the model described by Yokoyama et al. (1986) the hydrogel is composed of the three different kinds of phases: water phases (or PVA-poor phases), which in the frozen gel correspond to the crystal phases of ice; amorphous phases, in which every PVA chain is associated with water and PVA crystal phases, which restrain gross mobility of the amorphous chain. Repeated freezing and repeated melting (thawing) are both important; during repeated recrystallization (refinement process for ice crystals) growing ice crystals are excluding the polymer chains as impurity whenever the PVA-water system is frozen.

The turbidity of hydrogels was assessed by measuring transmittance of light by using ATR-FTIR (Supplementary Fig. 3). As can be seen, in case of 5% PVA the ~~decrease of~~ transmittance with increase of number of applied freeze-thaw cycles. In the case of 10% PVA, even the first cycle gives hydrogel with high turbidity (10% PVA hydrogel ~~with~~ 1 FT cycle was possible to take out of Petri dish as whole piece, in other words, to handle).

Since PVA hydrogel is formed by solely changing the temperature and no extra crosslinking agent was added, there is no usual chemical crosslinking inside of our hydrogels. Though, PVA crystallites are supposed to acted as junction points between polymer chains in the gel. The crystallinity level in hydrogels having different amount of freeze-thaw cycles (Supplementary Table 1) was assessed by method described in our manuscript (as a ratio~~n~~ of the absorption band in the range 1141-1145 ~~cm-1~~, which serves as an indicator of PVA crystallinity to the band at 1093 ~~cm-1~~, which assigned with ν(C-O) stretching vibrations). As can be seen from Supplementary Table 1, the ratio is increasing with increase of number of freeze-thaw cycles. It is expected, because each new cycle increases the PVA concentration in PVA-rich phase that means PVA chains approach each other easily and formation of junction between them is enhanced.

2 Stability of As-prepared Hydrogels

One way, which is used to assess a stability of hydrogel, is to immerse it in solvent (water). Swelling results of frozen gels prepared after different amount of freeze-thaw cycles are shown in Fig. 4. The frozen hydrogels were placed in 50 mL of fresh deionized water. Thawing (during 2 hours after gels were taken from freezer) are considered as swelling process. The swelling of as-prepared gels was conducted for 48 hours, same time as was used for samples (in manuscript) to reach equilibrium before compression test. Hydrogel made with 5% PVA and 1 freeze-thaw cycle was very weak (could not hold its own weight) and it was starting to dissolve after addition of 50 mL of deionized water.

In general, hydrogels made with 5% PVA were less stable than 10% PVA hydrogels. Swelling is slightly decreased with increase of number of freeze-thaw cycles that can indicate that crosslinking density is increasing. Moreover, similar to the results obtained by Abitbol et al. (2011), it was observed that CNC-containing PVA hydrogels were stable over the time, no disintegration was found along the cut edges when samples were submerged in water.

Results of compression test conducted on submerged in water samples (they were equilibrated in water for 48 hours prior compression test) presented in our manuscript (Table 5) show that gels possess the strength. It should be noticed that compression test conducted with samples in dry mode was not successful: integrity of hydrogel structure was destroyed by load while it was losing its water, while in wet mode the integrity of structure was preserved. It was also thought based on

previous experiments that freeze-drying (or lyophilic drying) can improve stability (long-term preservation) as well as mechanical properties of hydrogels (Butylina et al. 2016).

3 Supplementary Figures and Tables

3.1 Supplementary Figures

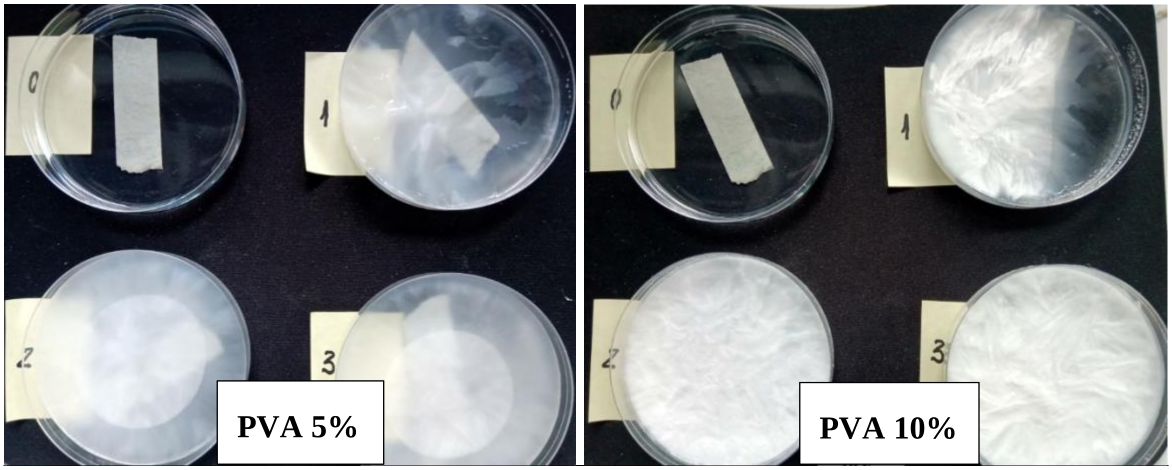

Supplementary Figure 1. Photo of the hydrogels obtained after certain amount of freeze-thawing cycles: from 0 to 3.

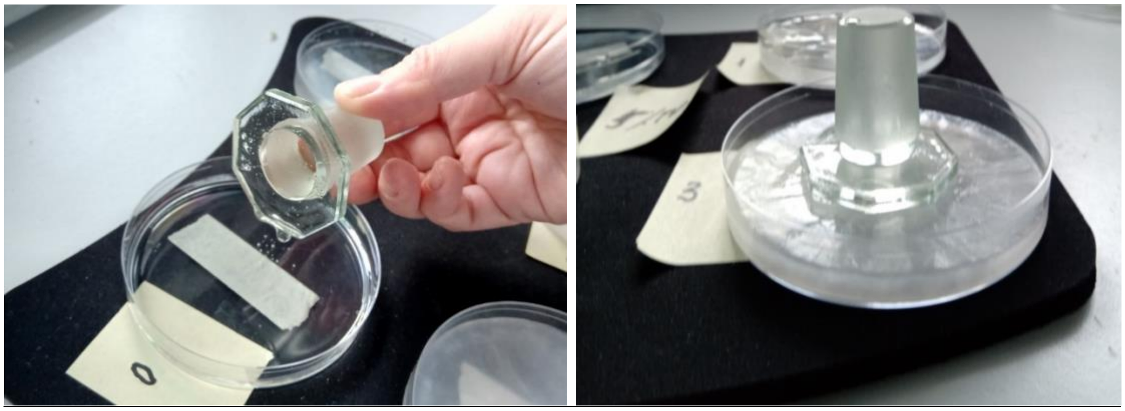

Supplementary Figure 2. Photo of hydrogels with 0 and 3 freeze-thaw cycles.

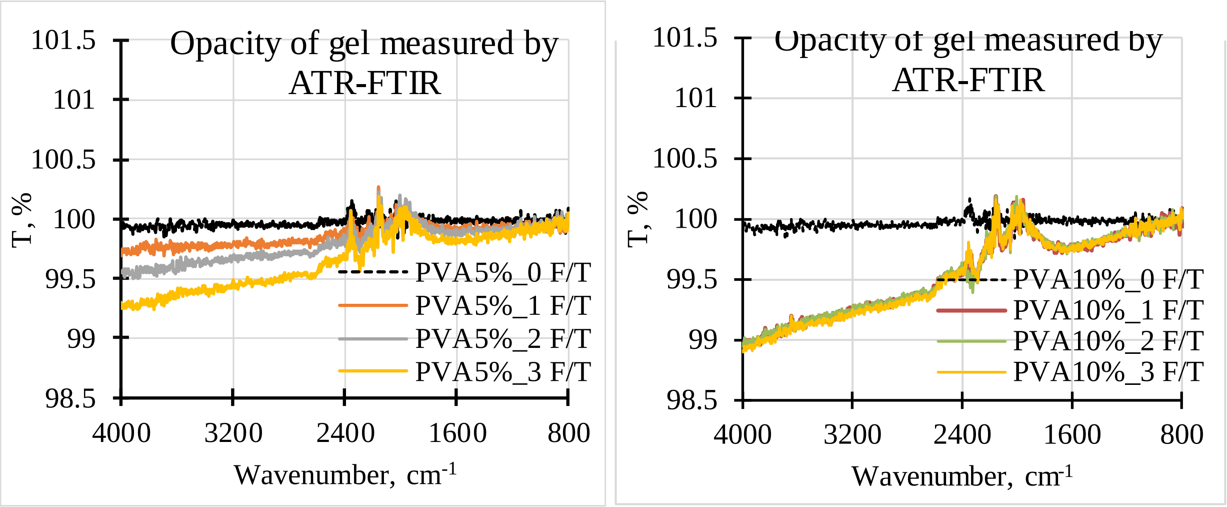

Supplementary Figure 3. ATR-FTIR spectra of 5% and 10% PVA hydrogels obtained after different freeze-thaw cycles.

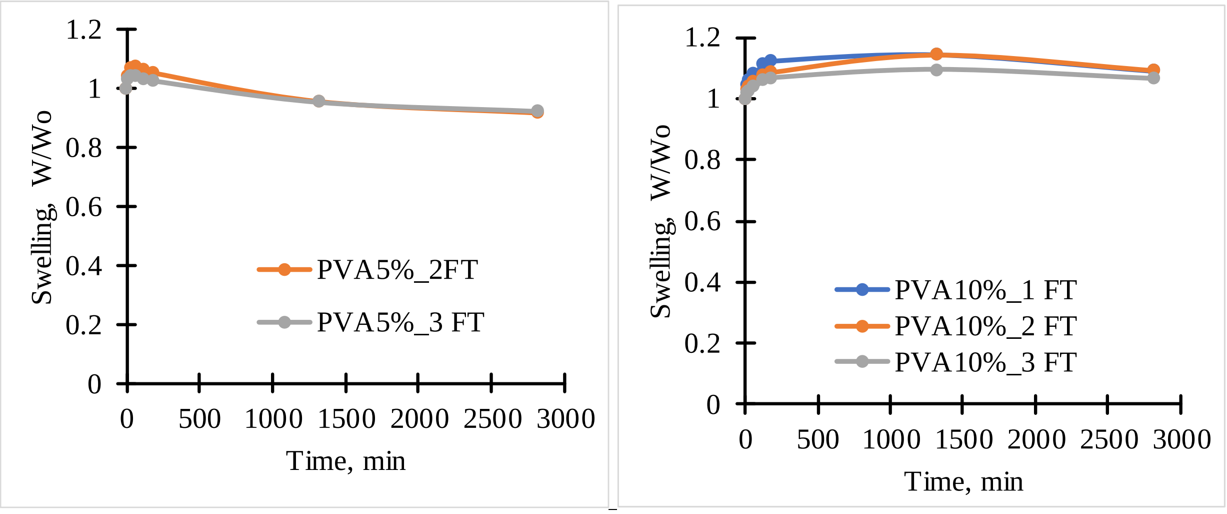

Supplementary Figure 4. Water uptake of frozen-thawed PVA hydrogels as a function of time. Hydrogels were prepared by freezing at – 20 °C for 24 hours and Wo is the initial frozen weight.

3.2 Supplementary Tables

Supplementary Table 1 Intensity of the crystallinity band at 1144 ~~cm-1~~ ~~was~~ normalized to the intensity of C-O stretching band at 1093 ~~cm-1~~

Sample	Number of freeze-thaw cycles	I_{1144}	I_{1093}	Ratio = I_{1144}/I_{1093}
5% PVA	1	0.007	0.016	0.428
	2	0.012	0.027	0.439
	3	0.015	0.031	0.477
10% PVA	1	0.022	0.046	0.482
	2	0.019	0.038	0.504
	3	0.035	0.066	0.536

References

Abitbol T, Johnstone T, Quinn TM, Gray DG. Reinforcement with cellulose nanocrystals of poly(vinyl alcohol) hydrogels prepared by cyclic freezing and thawing. Soft Matter (2011) 7:237379.

Butylina S, Geng S, Oksman K. Properties of as-prepared and freeze-dried hydrogels made from poly(vinyl alcohol) and cellulose nanocrystals using freeze-thaw techniques. Eur Polym J (2016) 81:386-96.

Yokoyama F, Masada I, Shimamura K, Ikawa T, Monobe K. Morphology and structure of highly elastic poly(vinyl alcohol) hydrogel prepared by repeated freezing-and-melting. Colloid Polym Sci (1986) 264:595-01.
